# Red‐Light‐Mediated Generation of Radicals: Applications in Organic Synthesis, Small‐Molecule Activation, Polymerization, and Bio‐Related Fields

**DOI:** 10.1002/anie.202501194

**Published:** 2025-06-29

**Authors:** Tong Zhang, Suman Pradhan, Shoubhik Das

**Affiliations:** ^1^ Department of Chemistry University of Antwerp Antwerp 2020 Belgium; ^2^ Department of Chemistry University of Bayreuth 95447 Bayreuth Germany

**Keywords:** Photodynamic therapy, Polymerization, Radicals, Red light, Synthetic methods

## Abstract

The field of visible light‐mediated photochemistry has experienced significant growth, leading to the development of a wide array of methodologies in synthetic organic chemistry. In particular, photocatalysis by using long‐wavelength light such as red and near‐infrared (NIR) light has garnered substantial attention. These strategies have inherent benefits of low energy, including minimal health hazards, less side reactions, and increased penetration through diverse reaction media. In this minireview, we present an overview of recent advancements in red‐ and NIR light‐induced photocatalysis for the generation of various radicals and key intermediates in organic synthesis. Additionally, this minireview will recount the application of small‐molecule activation, polymer science, and bio‐related aspects to offer a comprehensive framework and insight of photochemistry mediated by red and NIR light.

## Graphical abstract

Visible light‐driven photochemistry has advanced significantly, offering diverse methodologies in synthetic organic chemistry. Photocatalysis using red and near‐infrared (NIR) light is particularly notable due to its lower energy, reduced phototoxicity, minimized side reactions, and deeper penetration into reaction media. This minireview summarizes recent developments in red‐ and NIR‐mediated photocatalysis, emphasizing radical generation and reactive intermediates for organic synthesis. Applications in small‐molecule activation, polymer chemistry, and biologically relevant transformations are discussed, highlighting the growing potential of these photochemical processes in fundamental and applied chemistry.

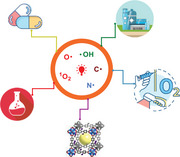

## 1 Introduction

Photoredox reactions have garnered significant interest due to their environmental benignity and adaptable applicability.^[^ ^1^, ^2^ ^]^ Within visible light‐mediated photocatalytic systems, active radicals are frequently generated, which exhibit high reactivity and play a significant role in various upgrading processes, ultimately leading to the valuable products.^[^ ^3^ ^]^

**Figure 1:**
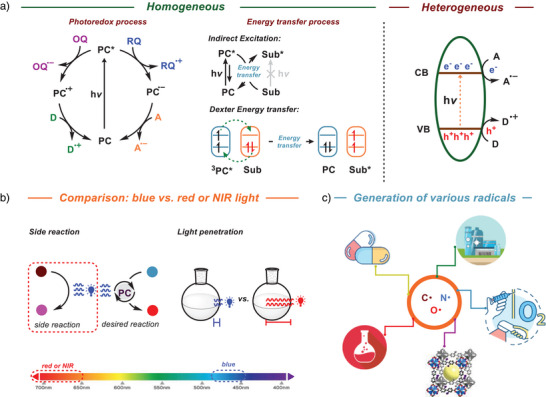
Mechanistic pathways for homo‐ and heterogeneous photocatalysis processes.

In general, to generate the target radicals, substrates or radical precursors are either reduced or oxidized by photocatalysts through redox processes or formed through energy transfer (EnT) pathways (Figure 1a).^[^ ^4^, ^5^ ^]^ In a homogeneous photoredox process, the photocatalyst is irradiated to its excited state after the absorption of light and this excited state of the photocatalyst, can be quenched through either an oxidative pathway or a reductive pathway.^[^ ^6^ ^]^ In both cases, the photocatalyst is regenerated from its corresponding reduced or oxidized form through interactions with suitable electron donors or acceptors. In addition to the photoredox process, photocatalysts can also activate substrates through the EnT process and in this process, upon irradiation by the light, the excited state of the photocatalyst transfers its energy to a substrate in the ground state, which otherwise remains inert to direct light activation.^[^ ^7^ ^]^ A variant of this process is the Dexter EnT where the excited donor facilitates electron transfer to the lowest unoccupied molecular orbital (LUMO) of the acceptor, while simultaneously receiving an electron from the highest occupied molecular orbital (HOMO) of the acceptor. This bidirectional electron exchange effectively transfers the excited‐state energy, leading to the formation of the excited state of the substrate and the regeneration of photocatalyst's ground state.^[^ ^8^ ^]^ In heterogeneous photocatalysis, a semiconductor is used, a material which conducts electricity more than an insulator, such as glass, but less than a pure conductor, such as metals.^[^ ^9^ ^]^ In solid‐state physics, the band gap (*E*_g_) generally refers to the energy difference (in electron volts) between the top of the valence band (**VB**) and the bottom of the conduction band (**CB**) (Figure 1a). The band gap energy is closely related to the HOMO/LUMO gap in chemistry. Substances with large band gaps are generally insulators, those with smaller band gaps are semiconductors, while conductors either have very small band gaps or none.^[^ ^10^ ^]^ When a photon with adequate energy is absorbed by the semiconductor, an electron from **VB** gets excited and jumps to the **CB**, which generates a hole in the **VB** and is known as the photoexcitation.^[^ ^9^ ^]^ The electrons on the **CB** move to the surface of the catalyst and participate in a reduction reaction, and the holes on the **VB** diffuse to the photocatalyst surface and allow the oxidation of organic molecules.

In general, these photoinduced reactions widely use transition metal‐based or organic photocatalysts, which are mainly activated under the irradiation of a blue light.^[^ ^11^ ^]^ In contrast to photochemical reactions facilitated by ultraviolet (UV) light, the utilization of less energetic blue light tends to increase selectivity and exhibit good to excellent functional group tolerance.^[^ ^12^ ^]^ However, blue light‐mediated photocatalytic processes still have certain constraints; for instance, blue light, situated within the higher‐energy region of visible light, remains susceptible to inducing inadvertent activation of photosensitive small‐molecule probes, resulting in the undesired side reactions.^[^ ^13^ ^]^ Furthermore, the scalability of the reaction under the irradiation of blue light proves to be challenging due to the limited penetration of light (Figure 1b).^[^ ^14^, ^15^ ^]^

These limitations have motivated researchers to explore longer‐wavelength regions, such as red light or near‐infrared (NIR) regions, since these wavelengths are associated with lower health risks, produce less side products due to their lower energy, and possess high penetration capabilities in solutions, facilitating reaction scalability.^[^ ^16^, ^17^ ^]^ In these longer‐wavelength regions, the photocatalysts are activated by the lower‐energy light, resulting in narrower redox windows. This allows for more precise control over chemical processes, enabling specific reactions to occur under well‐defined conditions. Furthermore, employment of low‐energy light‐mediated photocatalysis to generate radicals in the synthesis of chemicals and pharmaceuticals constitutes an attractive strategy in the aspect of chemical biology and biomedicine. Moreover, this approach aligns with the pursuit of sustainable development goals (SDGs), which are of paramount importance for our societies.^[^ ^18^ ^]^ Inspired by this, synthetic strategies based on red or NIR light‐induced photocatalysis have attracted much attention.^[^ ^19^ ^]^ This minireview explores diverse methodologies for generating key radicals, including carbon‐ and nitrogen‐centered radicals (Figure 1c). Furthermore, it covers the production of reactive oxygen species (ROS) and their applications in red‐light‐mediated photochemical processes, such as small molecule activation, polymerization, and bio‐related fields.^[^ ^20^ ^]^

## 2 The Design of Catalysts for Red‐Light‐Mediated Photocatalysis

**Figure 2:**
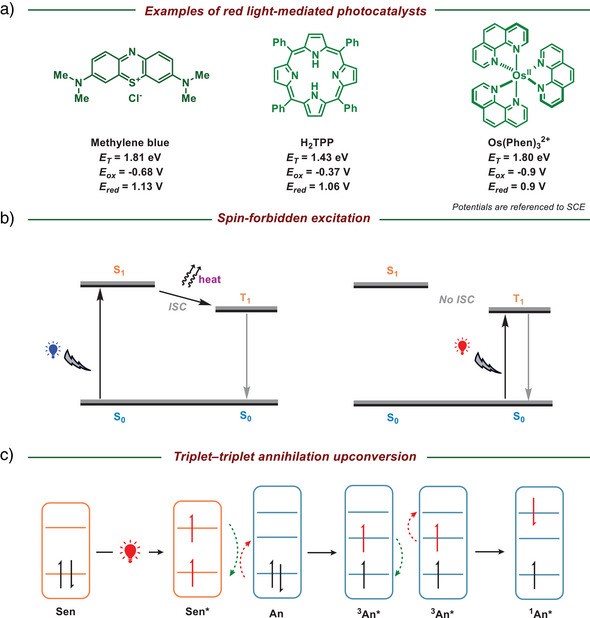
a) Representative red‐light‐mediated photocatalysts. b) Jablonski diagram of blue‐light‐induced excitation with ISC (left) and red‐light‐induced spin‐forbidden excitation (right). c) Jablonski diagram of triplet–triplet annihilation upconversion in red light photoredox catalysis.

The essential component in red light‐driven photochemical or photoredox reactions is the photosensitizer or photocatalyst. Radical generation can be generally categorized into two pathways: the direct and indirect approaches.^[^ ^4^, ^5^, ^6^ ^]^ In the direct pathway, chemical species are activated by photosensitizers through an energy transfer process, resulting in the formation of excited‐state species, such as reactive oxygen species, or the cleavage of weak chemical bonds, such as N─O bonds.^[^ ^5^ ^]^ Additionally, the indirect pathway involves the generation of target radicals via photoredox catalysis, wherein appropriate photocatalysts facilitate electron transfer processes.^[^ ^6^ ^]^ The efficiency of this reaction is governed by the compatibility between the redox potentials of the substrates and the catalysts. As previously discussed, red light‐activated photocatalysts are triggered by lower‐energy wavelengths (*λ* > 650 nm), corresponding to the energy gap between their HOMO and LUMO, leading to narrower redox potential windows compared to those activated by UV or blue light. Thus, modulating the energy gap between their HOMO and LUMO represents a key strategy in designing and synthesizing photocatalysts to be responsive toward red light. The extension of π‐conjugation, as exemplified in organic photocatalysts like methylene blue,^[^ ^21^ ^]^ aryl porphyrins,^[^ ^22^ ^]^ and polypyridyl complexes,^[^ ^23^ ^]^ reduces the HOMO–LUMO energy gap, thereby inducing a red shift in the absorption spectrum (Figure 2a). In general, blue light‐activated photocatalysts typically lose approximately 15−25 kcal mol^−1^ of energy as heat during intersystem crossing (ISC) from the singlet excited state (S_1_) to the triplet state (T_1_). To circumvent this energy dissipation, an alternative strategy for the design of red light‐activated photocatalysts employs spin‐forbidden excitation directly from the ground singlet state (S_0_) to the triplet state (T_1_), thereby avoiding the energy loss associated with ISC (Figure 2b).^[^ ^23^ ^]^ However, the limitations imposed by spin‐forbidden excitation restrict its practical applications. Spin‐forbidden electronic transitions exhibit low transition probabilities and weak spectral intensities due to spin selection rules, resulting in reduced quantum efficiency and extended excited‐state lifetimes.^[^ ^24^ ^]^ Overcoming these limitations often necessitates spin‐orbit coupling, which is typically enhanced by heavy atoms or intricate molecular design strategies.^[^ ^25^ ^]^ This is a fact that red light inherently activates only red‐light‐mediated photocatalysts; however, triplet–triplet annihilation upconversion (TTA‐UC) can produce more powerful catalysts that are not constrained by the energy of red light.^[^ ^26^ ^]^ The TTA‐UC process, illustrated by the Jablonski diagram, involves two key components: a sensitizer (Sen) and an annihilator (An). Upon absorption of red light, the photosensitizer transitions to its triplet excited state (T_1_). The sensitizer subsequently transfers its excitation energy to the annihilator, resulting in a ground‐state sensitizer and a triplet‐excited annihilator. When two triplet‐excited annihilators interact, they undergo triplet–triplet annihilation, generating a singlet excited state (S_1_) of the annihilator, which emits an upconverted photon to generate green or blue light (Figure 2c).^[^ ^26^ ^]^

In summary, to utilize red‐light‐mediated photoredox strategies, photocatalysts must absorb light within the 600–800 nm wavelength range, corresponding to a maximum energy threshold of ≤2.0 eV. Catalyst design strategies often focus on increasing π‐conjugation to enhance redshifted absorption or using low‐energy, spin‐forbidden excitations to minimize energy loss during ISC and directly activate the photocatalyst with low‐energy light. Additionally, efficient TTA‐UC can be employed, where various red‐light‐absorbing sensitizers, paired with corresponding annihilators, upconvert the initial low‐energy light source to emit higher‐energy light.

## 3 Generation of C‐Centered Radical Species via Red‐Light‐Mediated Photocatalysis

### 3.1 Aryl Radicals

**Figure 3:**
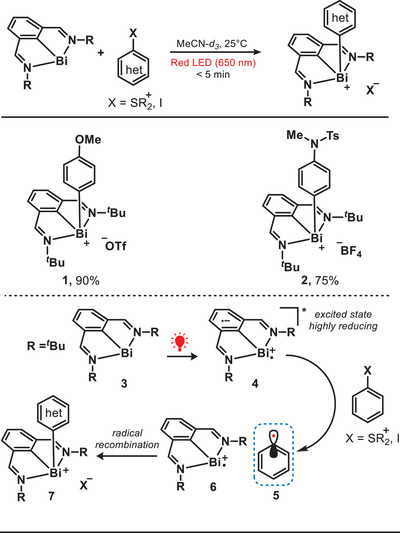
Red‐light‐mediated oxidative addition of aryl electrophiles.

Aryl radicals are considered highly versatile intermediates in organic synthesis, especially for facilitating functional group interconversion and the formation of carbon─carbon (C─C) bonds.^[^ ^27^ ^]^ These strategies predominantly utilized aryl halides^[^ ^28^ ^]^ or aryldiazonium salts^[^ ^29^ ^]^ as aryl precursors under the irradiation of blue light. Aryl halides or aryldiazonium salts typically undergo single‐electron reduction under blue light to generate the corresponding aryl radicals. Alternatively, red‐light‐based photoredox chemistry can also effectively promote radical generation from these precursors while minimizing undesired side reactions. In 2023, the group of Cornella reported a well‐designed organobismuth(I) complex to enhance the oxidative addition process of various aryl electrophiles under the irradiation of a red light (Figure 3).^[^ ^30^ ^]^ The photocatalyst *N*,*C*,*N*‐bismuthinidene (**3**) was successfully synthesized in a two‐step process involving the reaction of (1*E*,1′*E*)‐1,1′‐(2‐bromo‐1,3‐phenylene)bis(*N*‐*tert*‐butylmethanimine) with bismuth chloride. Upon the irradiation of a red light, the *N*,*C*,*N*‐bismuthinidenes reached the excited state (**4**) through a metal‐to‐ligand charge transfer (MLCT) process and the reduction potential of the excited state of *N*,*C*,*N*‐bismuthinidenes was determined to be approximately −1.79 V versus the standard calomel electrode (SCE). This high reduction potential demonstrated an efficient facilitation for the reduction of aryl electrophiles such as iodobenzene, thereby leading to the formation of key aryl radical (**5**). Later on, the aryl radical further recombined with the oxidized *N*,*C*,*N*‐bismuthinidenes to form the aryl‐bismuth(III) complex (**7**). This approach enabled the formation of diverse oxidative‐addition aryl‐bismuth complexes, providing a versatile platform for subsequent functionalization.

**Figure 4:**
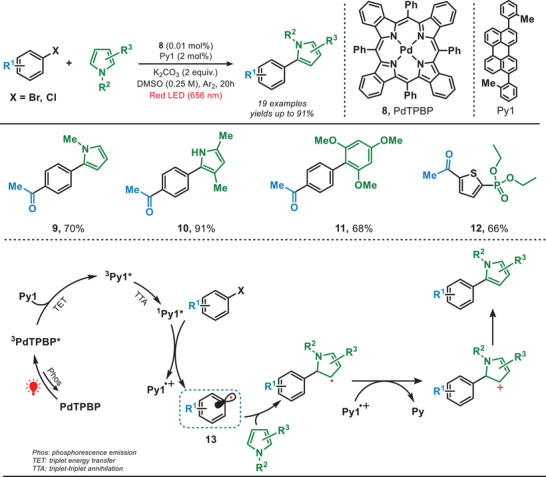
Red light‐driven reduction of aryl halides via a TTA strategy.

In general, triplet–triplet annihilation (TTA) has found extensive utility across various fields, including solar cells, light harvesting, organic light‐emitting diodes (OLEDs), and cancer therapy. In 2023, the group of Han reported a red‐light‐driven photocatalytic system for the reduction of aryl halides via TTA strategy by using meso‐tetraphenyltetrabenzoporphine palladium complex (PdTPBP) as a photosensitizer and perylene derivatives (Py) as reductants (Figure 4).^[^ ^31^ ^]^ In this TTA‐UC system, PdTPBP absorbed red light and reached the corresponding excited state (^3^PdTPBP*). This excited state subsequently transferred energy to Py1, promoting it to its singlet excited state ^1^Py1* through sequential triplet energy transfer (TET) and TTA pathway. The resulting ^1^Py1* efficiently reduced aryl halide to the corresponding aryl radical (**13**). Following this, the aryl radical successfully reacted with a range of pyrroles and polysubstituted benzenes, yielding the targeted products with high efficiency (**9**–**11**). Furthermore, the formation of carbon─phosphorus (C─P) bonds from heterocyclic ring systems (**12**) can also be achieved under these conditions. In this aspect, it is worth mentioning that the superior penetration capability of red light through solutions facilitated an effective large‐scale transformation under batch conditions compared to blue light‐mediated photoredox chemistry.

**Figure 5:**
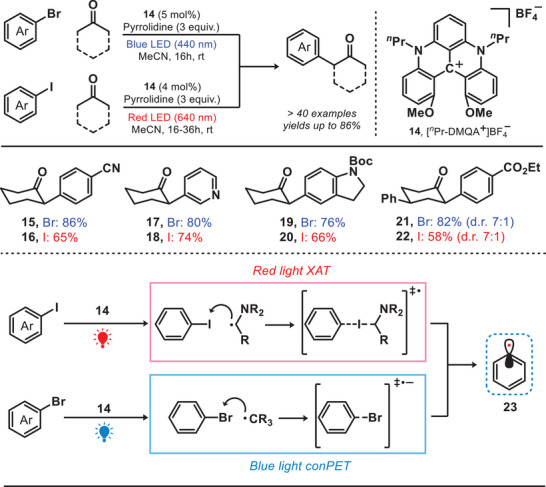
Photocatalytic system for chromoselective C(sp^2^)─*X* bond activation.

Inspired by this chemistry, the helical carbenium ion—dimethoxyquinacridinium ([*^n^* Pr‐DMQA^+^]BF_4_^−^) was introduced for the arylation reaction. In fact, *^n^* Pr‐DMQA^+^]BF_4_^−^ was well‐known to be activated under the irradiation of red light, has already been extensively studied and applied in various fields such as cascade trifluoromethylation and dearomatization.^[^ ^32^ ^]^ Recently, the research group of Gianetti reported a photocatalytic system by employing [*^n^* Pr‐DMQA^+^]BF_4_^−^ as a catalyst to facilitate chromoselective C(sp^2^)─*X* bond activation in multihalogenated aromatic compounds (Figure 5).^[^ ^33^ ^]^ Upon the irradiation of red light, the α‐amino radical was formed via an oxidative quenching pathway. This radical facilitated the abstraction of iodine atoms through a halogen atom transfer (XAT) process, resulting in the generation of crucial aryl radical intermediates (**23**).^[^ ^34^ ^]^ In contrast, under the irradiation of blue light, bromoarenes were reduced by the photocatalyst and generated the corresponding aryl radical through consecutive photoinduced electron transfer (conPET) process.^[^ ^35^ ^]^ The generation of aryl radicals can be selectively achieved through two distinct mechanisms by modulating the wavelength of the corresponding light source. For instance, in the red‐light‐mediated process, the bromo‐substituents remain unaffected and provide an additional opportunity for subsequent functionalization.

### 3.2 Alkyl Radicals

Alkyl radicals are recognized as essential and valuable intermediates which were extensively utilized in organic synthesis for constructing new bonds and synthesizing valuable chemicals.^[^ ^36^, ^37^ ^]^ Alkyl radicals are commonly generated via C─H functionalization or by utilizing various alkyl radical precursors such as alkyl halides. Advantageously, the use of red‐light‐induced photochemistry has the potential to broaden the range of application scenarios, thereby promoting advancements in both photoredox and photochemical methodologies.

#### 3.2.1 Reduction

Chlorophyll a (Chl a) has recently garnered significant attention due to its potential as a green photocatalyst.^[^ ^38^ ^]^ It is capable of absorbing a substantial amount of energy from violet‐blue and orange‐red wavelengths of light.^[^ ^39^ ^]^

**Figure 6:**
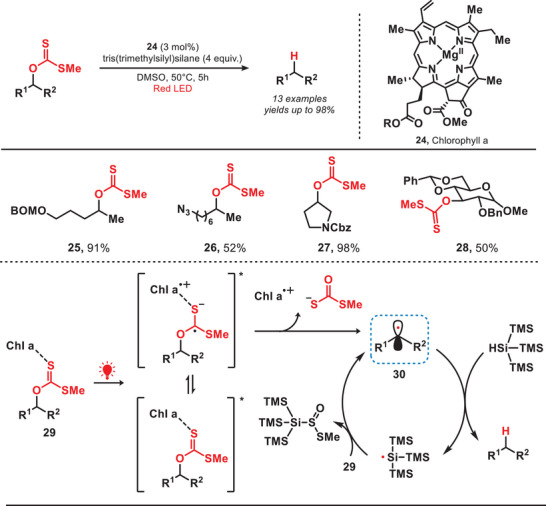
Red‐light‐induced Barton–McCombie reaction.

In 2020, Takao et al. developed a photocatalytic system for red‐light‐mediated Barton–McCombie reaction by using Chl a as a photocatalyst (Figure 6).^[^ ^40^ ^]^ In this reaction, Chl a initially formed the complex **29** with xanthate and subsequent activation by the red‐light‐induced electron transfer from Chl a to the xanthate resulted in the generation of key alkyl radical (**30**). The corresponding radical abstracted a hydrogen atom from tris(trimethylsilyl)silane (TTMSS), yielding the desired products and TTMSS radical, which later promoted the radical chain process. Using these methods, the deoxygenation of secondary long‐chain alcohols (**25** and **26**), cyclic alcohols (**27**), and bulky alcohols (**28**) can be effectively accomplished. Notably, this red‐light‐mediated approach circumvented the necessity of toxic organotin compounds and hazardous radical initiators, and in this way, enabled the Barton–McCombie reaction to proceed efficiently under milder reaction conditions.^[^ ^41^ ^]^

#### 3.2.2 Decarboxylation

**Figure 7:**
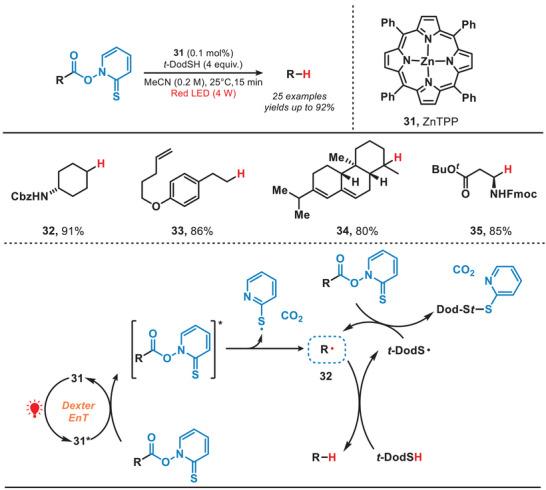
Photocatalytic Barton decarboxylation under the irradiation of red light.

In 2023, Ogura and coworkers developed a photocatalytic Barton decarboxylation method by employing zinc tetraphenylporphyrin (ZnTPP) as the photocatalyst, which operated under the irradiation of red light (Figure 7).^[^ ^42^ ^]^ The ZnTPP complex absorbed the red light, inducing Barton esters to reach an excited state through Dexter energy transfer (EnT), which resulted in the generation of alkyl radicals by following release of CO_2_ and sulfur‐centered radicals.^[^ ^43^ ^]^ The alkyl radical (**32**) subsequently abstracted a hydrogen atom from *t*‐dodecanethiol (*t*‐DodSH), yielding *t*‐dodecanethiol radical (*t*‐DodS•) and the desired decarboxylated product. Concurrently, the *t*‐DodS• radical further reacted with Barton esters, leading to the formation of alkyl radicals through a radical propagation process. Under mild reaction conditions, the decarboxylation of Barton esters containing diverse functional groups, including protected amino groups and terminal alkenes, proceeded efficiently without engaging in a photoredox mechanism. This enabled the tandem Giese‐type reaction to occur successfully under blue light irradiation, which expanded the application scope of Giese‐type reaction.

#### 3.2.3 Difunctionalization

**Figure 8:**
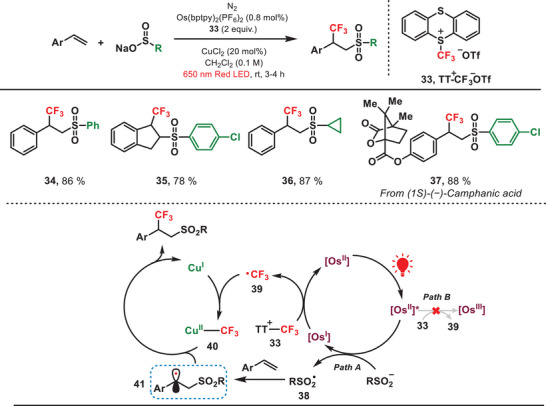
Red‐light‐induced regioselectivity switch in the difunctionalization of olefins.

Recently, our group has developed a red‐light‐mediated copper‐catalyzed sulfonyltrifluoromethylation of alkenes via the installation of a ─CF_3_ group at the α‐position to a phenyl ring (Figure 8).^[^ ^44^ ^]^ To achieve this regioselectivity switch, an osmium‐based photocatalyst, activated by low‐energy red light, was employed owing to its narrow redox potential window. This allowed the precise sequential formation of sulfonyl radical (RSO_2_•) and ─CF_3_ radical, governed by the thermodynamic parameters. The formed sulfonyl radical (**38**) underwent an addition reaction to alkene, leading to the generation of a carbon‐centered radical (**41**). Subsequently, the Cu(I) species captured the free ─CF_3_ radical (**39**), leading to the formation of the Cu(II)─CF_3_ complex (**40**), and finally, the cross‐coupling reaction between radicals **41** and **40** yielded the desired product. The scope of olefins and sulfinates, including both terminal and internal alkenes, as well as phenyl and aliphatic sulfinates, is extensive in production processes. It should be noted that this unique selectivity was not observed when utilizing a comparable blue‐light system because the ─CF_3_ radical was generated at a significantly faster rate than the sulfinyl radical. In addition, trifluoromethylation reagent (**33**) could be decomposed to generate CF_3_ radicals (**39**) via homolysis under the irradiation of blue light.^[^ ^45^ ^]^ Therefore, this red‐light‐mediated photocatalytic approach has successfully overcome the challenges related to the regioselective addition of radicals to alkenes.

## 4 Generation of N‐Centered Radical Species via Red‐Light‐Mediated Photocatalysis

Nitrogen‐centered radicals have garnered attention in the realm of photoredox reactions due to their versatility as intermediates in nitrogen‐containing chemical synthesis, which is imperative in drug designing and in the pharma research.^[^ ^46^, ^47^ ^]^ Nitrogen‐centered radical species exhibit high reactivity and are widely employed as potent hydrogen atom transfer (HAT) agents in C─H bond functionalization, including synthetic strategies utilizing the 1,5‐HAT process.^[^ ^48^ ^]^ Nitrogen‐centered radicals are typically generated through the cleavage of N─*X* bonds, such as N─H, N─Cl, N─S, N─N, or N─O, utilizing metal‐catalyzed or photocatalytic pathways.^[^ ^49^, ^50^ ^]^ The subsequent examples will illustrate the formation and advantage of nitrogen‐centered radicals generated via red‐light‐driven photochemistry in comparison to those produced via blue‐light photochemical processes.

**Figure 9:**
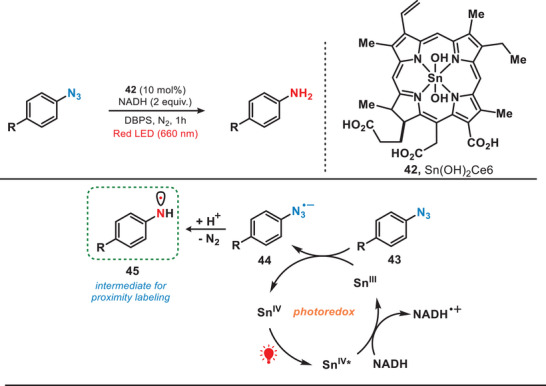
Red‐light‐induced proximity labeling by utilizing a tin‐chlorin complex.

Proximity labeling methodologies are advanced biochemical techniques used to study spatial and functional relationships between biomolecules.^[^ ^51^ ^]^ By employing engineered enzymes to tag nearby proteins or nucleic acids with identifiable labels, these methods enable the characterization of molecular interactions and dynamic biological phenomena within their native cellular environment.^[^ ^52^ ^]^ Nevertheless, prevailing approaches relied on the application of visible light, predominantly blue light; however, exhibited constraints in their application under intricate biological environments. To avoid this, in 2022, the group of MacMillan developed a red‐light‐mediated proximity labeling platform, known as *µ*Map‐Red, where a tin chlorin complex was used as a photocatalyst to convert phenyl azide biotin probes into their corresponding reactive nitrene or aminyl radical (Figure 9).^[^ ^53^ ^]^ Upon activation by the red light, Sn^IV^* was reduced to Sn^III^ in the presence of 1,4‐dihydronicotinamide adenine dinucleotide (NADH).^[^ ^54^ ^]^ The resulting Sn^III^ species facilitated the reduction of phenyl azide (**43**) to the corresponding phenyl azide radical anion (**44**). This radical anion subsequently underwent mesolytic cleavage and protonation, leading to the release of molecular nitrogen and the generation of reactive aminyl radical (**45**) for proximity labeling. Compared to the limited penetration depth of UV (<0.1 mm) and blue light (1–2 mm) in vivo, the Sn‐photocatalyst was activated at depths exceeding 10 mm because of the superior tissue penetration ability of red light. This activation facilitated the generation of reactive nitrene or aminyl radicals, enabling the photolabeling of proteins.

**Figure 10:**
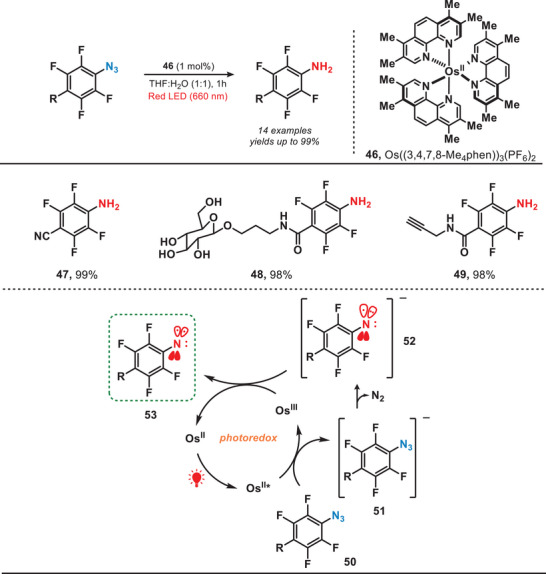
Red‐light‐induced proximity labeling utilizing an osmium complex.

Approximately at the same time, the research group of Rovis and colleagues reported a conceptually analogous strategy by involving the development of a red‐light‐activated osmium photocatalyst. The Os catalyst was employed to convert an aryl azide into triplet nitrene for proximity labeling (Figure 10).^[^ ^13^ ^]^ Upon exposure to red light, aryl azide (**50**) was reduced by Os^II^* to yield the reduced nitrene (**52**). Later, nitrene was further oxidized by Os^III^, resulting in the formation of triplet nitrene (**53**). This approach utilized a redox‐neutral electron transfer process dependent on both Os‐photocatalyst and red light, facilitating a photocatalytic system tailored for precise labeling applications. It is important to note that, in contrast to the formation of singlet nitrene through UV or blue light‐induced photolysis, this method exclusively generated triplet nitrene when both an Os‐photocatalyst and red light irradiation were employed. Therefore, due to this highly selective redox‐mediated azide activation, the system allowed the targeted delivery of the photocatalyst to specific cellular regions and enabled localized activation of aryl azide probes.

**Figure 11:**
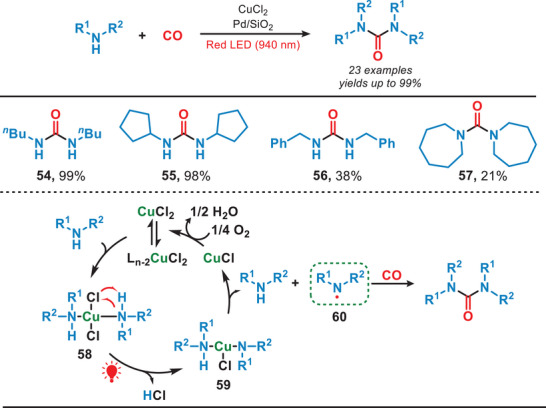
NIR‐mediated photocatalytic system for the carbonylation of amines.

In general, transition metal complexes (TMCs) typically require high‐energy UV–vis light for their activation due to the strong electron localization in the metal─ligand bonds. However, weak intermolecular interactions can induce aggregation into nanodots (NDs), enabling activation by lower‐energy red light. In 2022, Yuan et al. developed a photocatalytic system which was activated by the near‐infrared light for carbonylating amines with CO (Figure 11).^[^ ^55^ ^]^ They used uniformly dispersed TMC nanodots as heterogeneous photocatalysts and in this work, CuCl₂ dissolved in butylamine formed CuCl₂‐butylamine NDs (**58**). Red light irradiation promoted electron transfer, forming a nitrogen‐centered radical (**60**), which reacted with CO to yield the carbonylated amines. The TMC NDs exhibited remarkable efficiency in catalyzing the carbonylation of a wide range of amines, including both acyclic and cyclic amines, to form ureas under NIR irradiation. Compared to the utilization of UV or blue light, this study presented a promising approach for harnessing low‐energy photons, offering a pathway toward the efficient conversion of full‐spectrum solar energy into chemical energy.

## 5 Generation of Reactive Oxygen Species (ROS) via Red‐Light‐Mediated Photocatalysis

Reactive oxygen species (ROS) are highly reactive molecules that are primarily derived from oxygen or water through various chemical or biochemical processes.^[^ ^56^ ^]^ In practical applications, photoredox catalysis tends to exhibit a propensity for operation within an oxygen‐rich atmosphere or directly under aerobic conditions, particularly when necessitating clean oxidants.^[^ ^56^ ^]^ The rationale lies to activate oxygen molecule and to convert into diverse ROS, including superoxide anion radical (O_2_^•−^), hydrogen peroxide (H_2_O_2_), singlet oxygen (^1^O_2_), and hydroxyl radical (•OH).^[^ ^57^, ^58^, ^59^, ^60^ ^]^ These species serve as potent oxidants, facilitating oxidation and decomposition reactions.

### 5.1 Generation of Hydroxyl Radical

**Figure 12:**
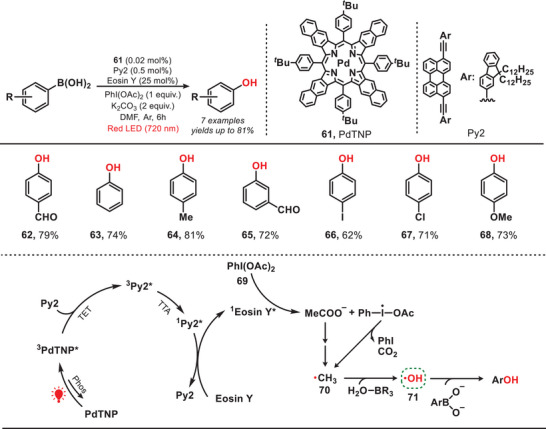
Photocatalytic oxidation of aryl boronic acid to aryl phenol under red light.

In 2020, the Han group developed a near‐infrared‐light‐mediated triplet–triplet annihilation upconversion (TTA‐UC) system by employing palladium tetraphenyltetranaphthoporphyrin (PdTNP) as a photosensitizer and perylene derivative (Py) as annihilator. This system effectively activated eosin Y as a photocatalyst, facilitating the oxidation of aryl boronic acid into aryl phenol (Figure 12).^[^ ^61^ ^]^ Upon excitation of eosin Y to its excited state (^1^eosin Y*), (diacetoxyiodo)benzene (PhI(OAc)_2_, **69**) underwent reduction, producing the methyl radical (**70**). The methyl radical abstracted the hydrogen atom from H_2_O‐BR_3_, resulting in the formation of the crucial hydroxyl radical (**71**). The hydroxyl radical then facilitated the oxidation of aryl boronic acid, ultimately yielding aryl phenol. This study reports the highest recorded NIR‐to‐green TTA‐UC efficiency (*Φ*_UC_ = 16.7% (653 nm)) achieved under conditions of low annihilator concentration and low‐power NIR excitation. This advancement significantly enhanced photocatalytic performance, even under ambient sunlight. The findings provide a versatile framework for developing highly efficient TTA‐UC systems with potential applications in photocatalysis, biomedical systems, and solar energy conversion.

### 5.2 Generation of Singlet Oxygen

**Figure 13:**
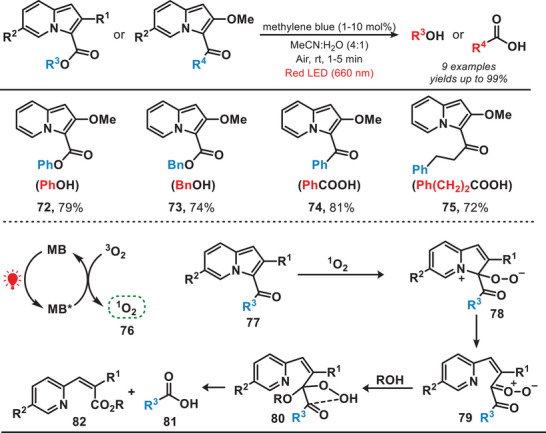
Red‐light‐induced photocatalytic system for the release of alcohols and carboxylic acids from 3‐acyl‐2‐methoxyindolizines.

Singlet oxygen demonstrates remarkable efficacy as a chemical species in effecting the cleavage of covalent bonds, particularly in catalyzing the oxidative cleavage of carbon═carbon double bonds.^[^ ^62^, ^63^, ^64^, ^65^ ^]^ In 2020, Hosoya et al. designed a red‐light‐activated photocatalytic system for the rapid release of alcohols and carboxylic acids from 3‐acyl‐2‐methoxyindolizine by utilizing methylene blue as a photosensitizer (Figure 13).^[^ ^66^ ^]^ In the photocatalytic system, the methylene blue, upon absorbing red light and transitioning to its excited state, converted triplet oxygen (^3^O_2_) into singlet oxygen (**76**). This singlet oxygen species subsequently facilitated the oxidation of 3‐acyl‐2‐methoxyindolizines (**77**) to an intermediate (**78**). Finally, this process led to the uncaging of diverse carboxylic acids or alcohols from 3‐acylindolizines. This approach enabled the efficient synthesis of phenol (**72**), benzyl alcohol (**73**), and carboxylic acids (**74**–**75**) from their 3‐acyl‐2‐methoxyindolizine precursors, achieving yields of up to 99%. Notably, this reaction was carried out using red light irradiation, which offers distinct advantages over high‐intensity light. Red light's lower phototoxicity and superior tissue penetration make it particularly well‐suited for applications in biological environments, enhancing both safety and effectiveness.

### 5.3 Generation of Superoxide Anion Radical

**Figure 14:**
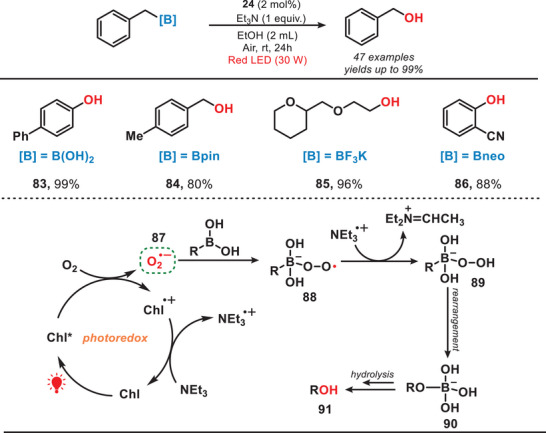
Red‐light‐mediated oxidative hydroxylations of organoborons.

As previously stated, chlorophyll as a green photocatalyst has found extensive applications in organic synthesis. Moreover, the synthetic procedure can effectively extract chlorophyll directly from natural sources such as spinach.^[^ ^67^ ^]^ In 2022, the group of Ouyang reported a red‐light‐mediated photocatalytic system for the oxidative hydroxylation reaction of organoboron compounds (Figure 14).^[^ ^67^ ^]^ They utilized spinach as the photocatalyst, leveraging chlorophyll within the spinach as the active photocatalytic agent. Upon the irradiation of light, O_2_ underwent reduction by the excited state of chlorophyll (ChI*) to generate superoxide anion radical (O_2_^•−^, **87**). This radical subsequently reacted with organoboron compound, forming organoboroperoxoate anion radical (**88**). Subsequently, hydroxylated product was obtained via sequential hydrogen abstraction, rearrangement, and hydrolysis reactions. This method demonstrated high efficiency and exhibited a broad substrate scope, successfully converting over 40 organoboron compounds into the corresponding hydroxylated products with yields reaching up to 99%. This method aligned closely with the principles of green chemistry, as it utilized chlorophyll directly extracted from green plant leaves, eliminating the need for toxic metals, synthetic dyes, or photocatalysts. Although large‐scale purification of chlorophyll may present a challenge in industrial applications. Additionally, ethanol, a low‐toxicity solvent, was employed, and in some cases, water served as a suitable solvent for specific substrates. Moreover, this protocol demonstrated compatibility with diverse organoboron reagents, including ─B(OH)₂, ─BF₃K, ─Bpin, and ─Bneo, enabling the synthesis of phenols, primary alcohols, secondary alcohols, and tertiary alcohols containing a wide range of functional groups.

## 6 Red‐Light‐Mediated Activation of Small Molecules

The use of lower‐energy light sources is not only appealing in organic synthesis but also offers significant benefits in the activation of small molecules, including H₂, CO₂, and water.^[^ ^68^ ^]^ In particular, red and NIR light offer mild reaction conditions and demonstrate exceptional tolerance to functional groups. Additionally, the intrinsic properties of lower‐energy light allow for superior penetration into reaction media compared to UV or blue light, making it especially suitable for large‐scale small molecule activation systems.

### 6.1 H_2_ Evolution

**Figure 15:**
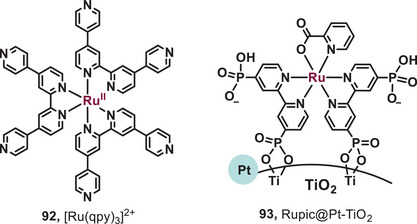
Red‐light‐activated photocatalysts for H_2_ evolution.

Hydrogen has gained recognition as a clean energy source due to its ability to power fuel cells, producing electricity and energy while generating only water as a by‐product.^[^ ^69^ ^]^ Among the diverse strategies for H₂ production, photochemical processes, which convert solar energy into chemical energy (e.g., H₂), are particularly promising because these methods align with the principles of green chemistry and sustainable economy, as they minimize waste generation.^[^ ^69^ ^]^ Commonly utilized photosensitizers, such as [Ru(bpy)₃]^2^⁺, are limited to absorbing light with wavelengths below 560 nm, which reduces their efficiency in solar energy conversion. To address this limitation, π‐extension of the ligand is employed in metal‐based photosensitizers to enhance their ability to absorb a broader range of the visible light spectrum.^[^ ^70^ ^]^ In 2015, the group of Hanan reported a ruthenium quaterpyridine complex as a red light‐driven photocatalyst for H_2_ evolution reaction (Figure 15, **92**).^[^ ^71^ ^]^ The ruthenium quaterpyridine complex ([Ru(qpy)_3_]^2+^) was feasibly synthesized through microwave irradiation in 15 min. Utilizing [Ru(qpy)_3_]^2+^ as a photosensitizer, the turnover number (TON) for H_2_ evolution approached approximately 360 under the irradiation of 630 nm light, whereas [Ru(bpy)₃]^2^⁺ exhibited a significantly lower TON of around 25. In addition, in 2023, Kobayashi et al. developed a red‐light‐induced ruthenium‐picolinate photosensitizer for water reduction and H_2_ evolution (Figure 15, **93**).^[^ ^72^ ^]^ The complex Et_3_NH[Ru(H_3_dpbpy)_2_(2‐pic)] (Rupic) was immobilized on the surface of platinum‐doped titanium dioxide (Pt‐TiO_2_) to synthesize the photosensitizer Rupic@Pt‐TiO_2_. Due to the better light absorption, the photosensitizer Rupic@Pt‐TiO_2_ can stably generate H_2_ under red light irradiation. Using Rupic@Pt‐TiO_2_ as a photosensitizer, the TON for H_2_ evolution reached ca. 400 under irradiation with 630 nm light.

### 6.2 CO_2_ Reduction

**Figure 16:**
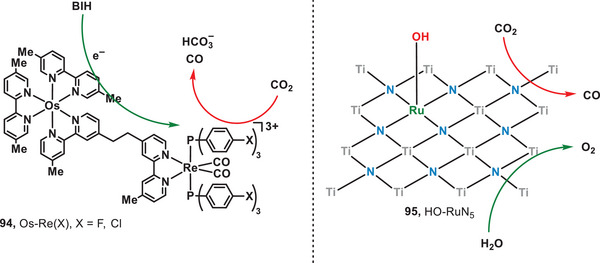
Red‐light‐induced supramolecular complexes for CO_2_ reduction.

The rapid urbanization and human activities have significantly increased atmospheric carbon dioxide (CO_2_) concentrations, resulting in the global warming, extreme weather, and associated environmental challenges.^[^ ^73^, ^74^ ^]^ Therefore, the advancement of efficient decarbonization technologies for the capture, storage, and utilization of CO_2_ presents a promising approach to addressing these challenges. Recently, photocatalytic CO_2_ reduction systems have been extensively studied and developed, as the conversion of solar energy into chemical energy represents an optimal approach for CO_2_ utilization.^[^ ^75^, ^76^, ^77^ ^]^ However, most existing strategies primarily rely on UV or blue light, which utilize only a narrow portion of the solar spectrum. Consequently, advancing CO_2_ reduction technologies that harness long‐wavelength light sources is essential.^[^ ^78^ ^]^ In 2013, Ishitani and coworkers engineered on supramolecular complexes (Os‐Re(*X*), *X* = F, Cl; **94**) integrated through Os(II)‐complexes and Re(I)‐complexes to catalyze the reduction of CO_2_ under the irradiation of red light (Figure 16, left).^[^ ^79^ ^]^ Within the supramolecular catalyst, Os(II)‐complex and Re(I)‐complex acted as photoredox catalysts. Upon light irradiation, 1,3‐dimethyl‐2‐phenyl‐2,3‐dihydro‐1*H*‐benzo[*d*]imidazole (BIH) was oxidized by the Os(II)‐complex and the electron was subsequently transferred to the associated Re(I)‐complex, and finally, CO_2_ was reduced by the Re(I)‐complex to CO and bicarbonate (HCO_3_^−^). The highest turnover number reached 1138 with Os‐Re(Cl). In 2023, Wang et al. developed a metallic photocatalyst, hydroxyl‐bonded ruthenium on titanium nitride surface (HO‐Ru/TiN, **95**), for red‐light‐induced CO_2_ reduction with H_2_O (Figure 16, right).^[^ ^80^ ^]^ In the catalytic system, the pivotal Lewis pair comprising HO─RuN_5_─Ti, facilitated by the hydroxyl group bonded on the surface, played a critical role in catalyzing the activation of CO_2_. To elucidate the role of HO─Ru species, the photocatalytic reduction of CO₂ was performed using pristine TiN, achieving a CO production rate of 6.64 µmol g⁻¹ h⁻¹. Upon the incorporation of Ru species, the CO production rate significantly increased to 69.09 µmol g⁻¹ h⁻¹. This study demonstrated the significant promise of metallic materials for catalyzing both the reduction of CO_2_ and the oxidation of water under low‐intensity light irradiation.

### 6.3 Water Splitting

**Figure 17:**
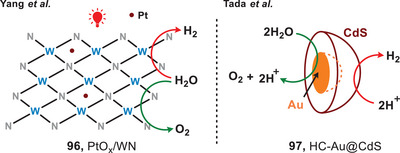
Red‐light‐activated photocatalysts for water splitting.

Given the significance of H₂ evolution highlighted in the previous section, the use of photocatalysts for water splitting represents a simple and optimal approach to hydrogen production. This method is characterized by high atomic efficiency, with oxygen as the sole byproduct.^[^ ^81^ ^]^ In 2017, the group of Yang developed a metallic photocatalyst made of black tungsten nitride doped with platinum (PtO*_x_*/WN) (**96**), which was capable of overall water splitting under the irradiation of red light (Figure 17, left).^[^ ^19^ ^]^ Under the irradiation of 600 nm light, ca. 1.0 nmol•h^−1^ H_2_ and 0.5 nmol•h^−1^ O_2_ were obtained from pure water. While the photocatalytic efficacy of water splitting remains low, this study showed the prospective utilization of metallic materials within this field. Later, in 2018, Tada et al. reported an Au–CdS half‐cut nanoegg photocatalyst (**97**) as a heteronanostructured (HNS) photocatalyst for red‐light‐mediated water splitting (Figure 17, right).^[^ ^82^ ^]^ It featured a core composed of Au nanoparticles enveloped by a shell of CdS material, resembling the structure of a half‐cut egg. In the catalytic system, Au nanoparticles facilitated the oxidation of water, resulting in the evolution of oxygen and protons, while the CdS shell promoted hydrogen evolution. The quantum yield of water splitting using this Au‐CdS half‐cut nanoegg photocatalyst under red light irradiation reached 0.24% and the rate of H_2_ generation reached ca. 79.2 µmol g⁻¹ h⁻¹.

### 6.4 COF‐, MOF‐, or CMP‐Catalyzed Aerobic Oxidation

#### 6.4.1 Covalent Organic Framework (COF)

**Figure 18:**
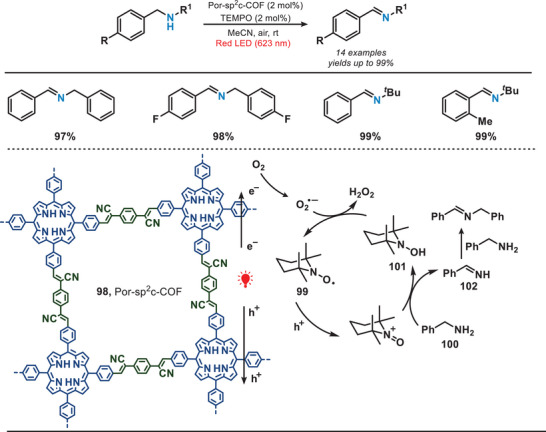
COF‐catalyzed aerobic oxidation of benzylamines under red light irradiation.

In 2020, the Lang group reported the synthesis of a COF featuring a 2D sp^2^ carbon‐conjugated porphyrin (Por) units, which was employed for the aerobic oxidation of benzylamines (Figure 18).^[^ ^83^ ^]^ The Por‐sp^2^c‐COF (**98**) was prepared from 5,10,15,20‐tetrakis(4‐benzaldehyde)porphyrin (*p*‐Por‐CHO) and 1,4‐phenylenediacetonitrile (PDAN) via the Knoevenagel condensation reaction.^[^ ^84^ ^]^ The π‐conjugation within the porphyrin linker facilitated a significant enhancement in the absorption of red light. Due to the optimal molecular size of TEMPO, it exhibited versatile positioning capabilities, allowing it to reside either externally or internally within the pores of Por‐sp^2^c‐COF.^[^ ^85^ ^]^ Upon the irradiation of red light, electron‐hole pairs were generated and separated within the Por‐sp^2^c‐COF. TEMPO (**99**) underwent oxidation facilitated by the COF, leading to the capture of the benzylic hydrogen from benzylamine (**100**), resulting in the formation of TEMPOH (**101**) and an imine intermediate (**102**). This intermediate subsequently engaged in a reaction with benzylamine to yield the desired product. This 2D COF facilitated a highly selective reduction of amines to imines (yields are up to 99%) in conjunction with the photocatalyst TEMPO, which is rarely observed in amorphous polymers or molecular dye systems.

#### 6.4.2 Polymeric Carbon Nitride (PCN)

**Figure 19:**
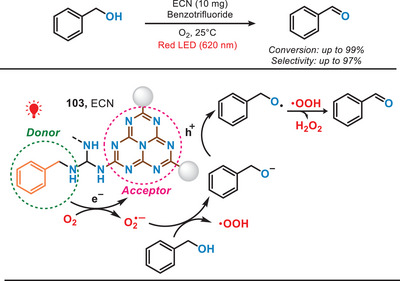
Photocatalytic selective aerobic oxidation of benzyl alcohols under red light.

In 2021, Wang et al. modified the PCN by incorporating biomimetic donor–acceptor motifs, enabling selective aerobic oxidation of benzyl alcohols under the irradiation of red light (Figure 19).^[^ ^86^ ^]^ The synthesis process began with the pyrolysis of melamine to produce PCN, followed by treating bulk PCN with molten salt to generate molten salt‐treated carbon nitride (MCN). Finally, MCN underwent photochemical modification to prepare the ECN (extended π‐conjugated PCN) catalyst. The enhanced π‐conjugation system and effective electron−hole charge carrier separation in the well‐established ECN enabled efficient oxidation of benzyl alcohols with high selectivity under the irradiation of red light.^[^ ^87^ ^]^ In addition to the oxidation of alcohols, benzylic C─H oxidation and selective oxidation of diphenyl sulfide can also be performed to produce valuable ketones and sulfoxides. This work also offered a novel surface engineering technique, characterized by its simplicity and efficacy, for the fabrication of precisely defined and exceptionally efficient photocatalysts.

#### 6.4.3 Metal‐Organic Framework (MOF)

**Figure 20:**
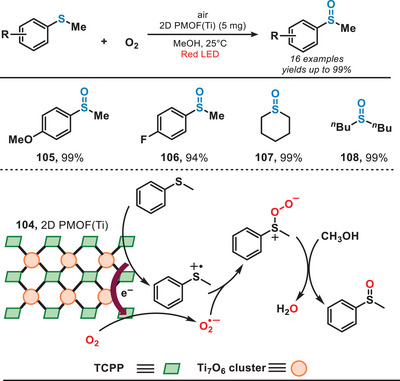
MOF‐catalyzed aerobic oxidation of sulfides.

In 2022, the group of Lang introduced a novel class of 2D titanium‐based MOFs for the photocatalytic aerobic oxidation of sulfides under the irradiation of red light (Figure 20).^[^ ^88^ ^]^ This MOF catalyst was synthesized through a process involving a mixture of benzoic acid, H_2_TCPP (4,4,4,4‐(porphine‐5,10,15,20‐tetrayl)tetrakis(benzoic acid)), and Ti(OBu)_4_, utilizing sequential sonication, stirring, and hydrothermal treatment. Due to the enhanced photoelectric characteristics and increased availability of active sites, sulfides were oxidized to form sulfoxides with exceptional conversion and selectivity (>98%). Within this system, sulfides underwent oxidation to produce the corresponding sulfur radical cations (RS^•+^). Meanwhile, atmospheric oxygen served as the primary source of oxygen, undergoing reduction to O_2_^•−^. Subsequently, sulfoxide was formed in the presence of methanol. This method enabled the efficient conversion of various sulfides, including aryl, alkyl, and cyclic sulfides, into their corresponding sulfoxides, achieving yields of up to 99% and selectivity reaching 100%. Due to the lower energy of red light, this system demonstrated improved functional group tolerance, such as iodophenyl sulfides, compared to photoredox strategies mediated by UV or blue light.

#### 6.4.4 Conjugated Microporous Polymer (CMP)

**Figure 21:**
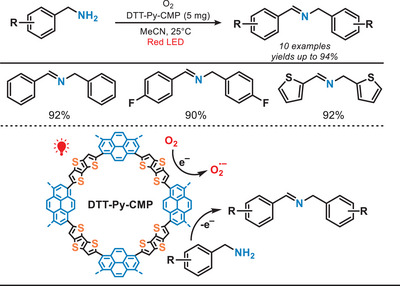
CMP‐catalyzed oxidation of amines under the irradiation of red light.

In 2023, Lang et al. reported conjugated microporous polymers (CMPs) for selective oxidation of amines under the irradiation of red light (Figure 21).^[^ ^89^ ^]^ The synthesis of the polymer DTT‐Py‐CMP involved the reaction of dithieno[3,2‐*b*:2′,3′‐*d*]thiophene (DTT) and 1,3,6,8‐tetrabromopyrene (Py) through C─H arylation.^[^ ^90^ ^]^ Under red‐light irradiation, this photocatalyst facilitated the oxidation of benzylamines, including both primary and secondary amines, achieving good conversion (up to 95%) and exceptional selectivity (>99%). Owing to the mild reaction conditions and the use of a lower‐energy light source, the system demonstrated excellent tolerance toward various functional groups while avoiding the over‐oxidation of products.

## 7 Red‐Light‐Mediated Polymerization

In addition to the application in organic synthesis, red‐light‐mediated photoredox reactions have found extensive utility in diverse fields, such as controlled radical polymerizations (CRP).^[^ ^91^, ^92^ ^]^ By toggling the light source on and off, visible light‐mediated CRP enables precise spatial and temporal control over polymerization processes, facilitating the synthesis of well‐defined polymers. In this aspect, Lacôte, Lalevée, and coworkers developed a strategy of emulsion photopolymerization of methyl methacrylate(MMA) using methylene blue (MB)/sodium *para*‐toluenesulfinate (NapTS) system under red light irradiation.^[^ ^93^ ^]^ This approach holds potential for diverse applications, including biomedical advancements and printing technologies.^[^ ^94^ ^]^

### 7.1 Reversible Addition–Fragmentation Chain Transfer (RAFT)

**Figure 22:**
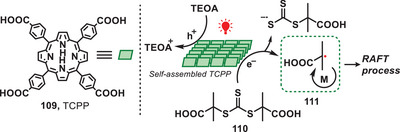
Red‐light‐induced polymerization via RAFT.

Reversible addition‐fragmentation chain transfer (RAFT) is a controlled radical polymerization technique that uses chain transfer agents to regulate polymer growth. This process enables precise control over molecular weight and composition, making it versatile for synthesizing well‐defined polymers for applications in fields like materials science, biomedicine, and nanotechnology.^[^ ^95^ ^]^ In 2020, Qiao et al. pioneered the synthesis of a metal‐free photocatalyst, which was used for facilitating controlled radical polymerization under the irradiation of red or near‐infrared light in aqueous conditions (Figure 22).^[^ ^96^ ^]^ The novel self‐assembled carboxylated porphyrin (SA‐TCPP) photocatalyst was synthesized via the chemical transformation of 5,10,15,20‐tetra(4‐carboxyphenyl)porphyrin (TCPP, **109**). This involved successive steps of deprotonation, neutralization, dialysis, and drying, resulting in the formation of rod‐like photocatalyst. Upon the irradiation of light, electron−hole charge carriers were separated and trithiocarbonate (**110**) was reduced to carbon‐centered radical (**111**), which subsequently formed a well‐defined polymer via RAFT process. It is important to emphasize that this methodology demonstrated an enhanced capacity for polymerization under aqueous conditions, exhibiting an accelerated polymerization rate compared to systems utilizing alternative light‐mediated photocatalysts.

#### 7.1.1 Atom Transfer Radical Polymerization (ATRP)

**Figure 23:**
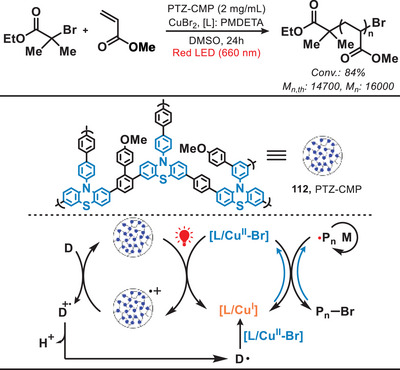
Red‐light‐activated heterogeneous catalytic system for ATRP.

Similar to RAFT, Atom transfer radical polymerization (ATRP) is also a controlled radical polymerization technique, which involves the reversible activation and deactivation of growing polymer chains through a redox process mediated by a transition metal catalyst and an alkyl halide initiator.^[^ ^97^ ^]^ In 2021, Matyjaszewski and coworkers presented a green‐ or red‐light‐mediated photocatalytic system which enabled copper‐catalyzed ATRP with conjugated microporous polymers as heterogeneous photocatalysts for controlled radical polymerizations (Figure 23).^[^ ^97^ ^]^ The photocatalyst PTZ‐CMP (**112**) was engineered through the synthesis of phenothiazine (PTZ) as the photoactive core, employing dimethoxybenzene as a cross‐linking agent via the Friedel–Crafts reaction. In this Cu‐catalyzed ATRP, irradiation by light induced the separation of electron−hole charge carriers within PTZ‐CMP and electrons from PTZ‐CMP facilitated the reduction of [L/Cu^II^‐Br] to form the ATRP activator [L/Cu^I^].^[^ ^98^, ^99^, ^100^, ^101^ ^]^ In addition, amines, acting as electron donors, underwent oxidation by PTZ‐CMP radical cation to generate the amine radical cation (D^•+^). This species then promoted the formation of *α*‐aminoalkyl radical (D•), which subsequently reacted with [L/Cu^II^‐Br], leading to the generation of [L/Cu^I^]. This further facilitated the efficient and well‐controlled polymerization of acrylate and methacrylate monomers. Notably, compared to other Cu‐catalyzed light‐mediated homogeneous systems (which often utilize UV or blue light), this heterogeneous photocatalyst exhibits the advantages of easy separability and the ability to maintain high photocatalytic efficiency for polymerization even after multiple recycling cycles. Subsequently, in 2023, the same research group disclosed the inaugural instance of fully oxygen‐tolerant red/NIR‐light‐mediated photoinduced atom transfer radical polymerization (photo‐ATRP) conducted in a high‐throughput fashion under biologically pertinent conditions, utilizing commercially available methylene blue (MB^+^) as the photosensitizer and the [X−Cu^II^/TPMA]^+^ (where TPMA denotes tris(2‐pyridylmethyl)amine) complex as the deactivator.^[^ ^102^ ^]^

#### 7.1.2 Reversible Deactivation Radical Polymerization (RDRP)^[^ ^103^ ^]^

A newly established category of radical polymerization is reversible deactivation radical polymerization (RDRP), which has attracted interest in 3D printing technologies, especially in macroscale vat‐photopolymerization methods like digital light processing (DLP). In contrast to traditional free radical photopolymerization, photoinduced RDRP facilitates the creation of 3D‐printed items with live attributes, permitting alterations after manufacturing. Although two‐photon polymerization‐based direct laser writing (3D DLW) is a well‐established method for creating intricate 3D microstructures, the utilization of reversible‐deactivation radical polymerization (RDRP) and its related benefits at this scale is still insufficiently investigated.

In response to this research deficiency, Spangenberg et al. explored an RDRP‐based approach to create a photoresist suitable for 3D DLW, employing nitroxide‐mediated photopolymerization (NMP2) to fabricate 4D microstructures.^[^ ^104^ ^]^ Their methodology utilized two‐photon‐induced nitroxide‐mediated polymerization to produce reactivable polymers during the 3D production process. The research exhibited the efficacy of an alkoxyamine‐based photoresist in two‐photon polymerization over a wide spectrum of wavelengths. The impacts of laser power and excitation wavelength on fabrication and postmodification were extensively analyzed. The findings demonstrate that controlling these factors enables fine control over photoinduced changes at nano‐ to micrometer scales. This study offers essential insights for enhancing the implementation of RDRP in 3D DLW and, more generally, in photopolymerization‐based 3D printing, thereby paving the way for future research in the domain.

## 8 Red‐Light‐Mediated Photodynamic Therapy

**Figure 24:**
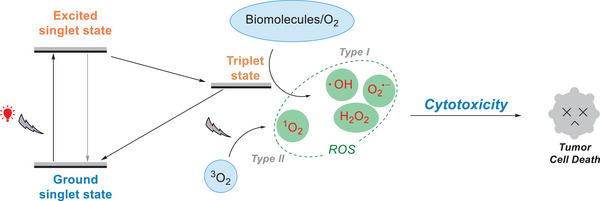
Red‐light‐triggered photodynamic therapy.

Numerous therapeutic approaches, such as surgery, chemotherapy, and radiation therapy, have been developed in the quest to cure cancer.^[^ ^105^, ^106^ ^]^ However, achieving selective cytotoxicity, where cancer cells are eliminated while preserving healthy cells, remains a significant challenge in oncological treatment.^[^ ^107^ ^]^ Photodynamic therapy (PDT) has attracted much attention and is applied for tumor treatment, as it can overcome this issue due to its noninvasiveness, high selectivity, and minimal side effects. In the PDT treatment, two primary processes lead to the generation of ROS. In the Type I pathway, ROS such as O_2_^•−^, H_2_O_2_, and •OH are produced via electron transfer from oxygen and biomolecules.^[^ ^108^ ^]^ Alternatively, in the Type II pathway, ^1^O_2_ is generated via energy transfer.^[^ ^109^ ^]^ Both processes effectively produce ROS, which are crucial to induce cytotoxic effects to kill tumor cells (Figure 24).^[^ ^110^ ^]^

### 8.1 Type I PDT

**Figure 25:**
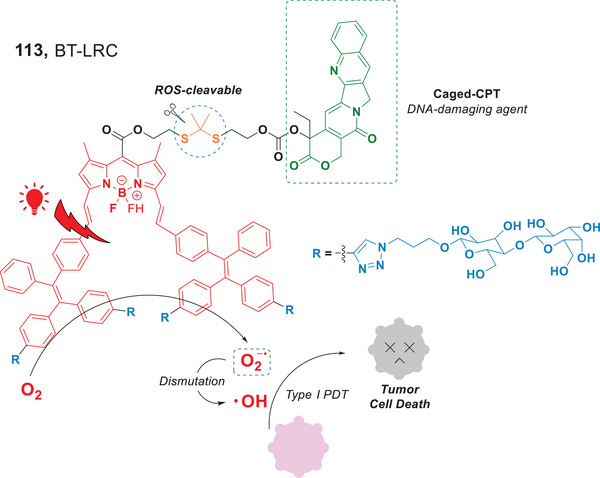
Red light‐induced synergistic Type I PDT and CPT chemotherapy.

Recently, the Xing group developed a nanoscale prodrug, BT‐LRC (**113**), a lactosylated photocage that covalently incorporated camptothecin (CPT) (Figure 25).^[^ ^111^ ^]^ This nano‐prodrug demonstrated the ability to produce O_2_^•−^ via Type I PDT upon irradiation by a red light source. These reactive oxygen species underwent dismutation to generate •OH, and both contributed to the destruction of tumor cells. Additionally, CPT covalently linked to the prodrug via ROS‐cleavable thioketal bonds, was released as a DNA‐damaging agent. As a result, this nano‐prodrug exhibited a synergistic therapeutic effect in cancer treatment. Their research has demonstrated the potential of photocage‐based glycosylated nanoparticles for the control of drug release and Type I PDT.

### 8.2 Type II PDT

**Figure 26:**
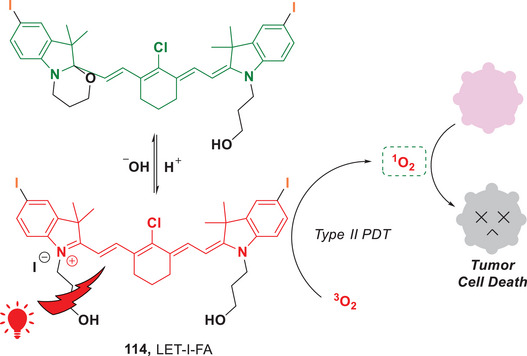
A pH/light dual‐controllable Type II PDT for cancer treatment.

In addition to advancements in type I PDT, significant progress has been made in the development of type II PDT. Recently, the Lin group introduced a dual‐controllable PDT process by using cyanine‐based long‐wavelength photosensitizers (Figure 26).^[^ ^112^ ^]^ These photosensitizers (PSs), such as LET‐I‐FA (**114**) were selectively activated within the acidic tumor microenvironment. Upon irradiation with red or NIR light, these PSs efficiently produced ^1^O_2_ via an energy transfer process, inducing tumor cell apoptosis. Importantly, this type II PDT approach is capable of operating with low‐energy light irradiation, which minimizes photothermal effects on the patient's skin. This reduction in thermal stress may alleviate acute pain and enhance the clinical feasibility of the treatment. Significantly, for in vivo applications, the probe LET‐I‐FA demonstrates a specific response to the acidic tumor microenvironment, enabling high‐resolution activatable fluorescence/photoacoustic molecular imaging. This study presents an innovative approach for screening activatable photosensitizers, thereby facilitating molecular imaging‐guided PDT.

## 9 Summary and Outlook

As elucidated in each section, the strategies for generating various crucial radicals under red or NIR light irradiation demonstrated significant potential for long‐wavelength light‐mediated photocatalysis in organic synthesis and the activation of small molecules. In this minireview, we have presented a summary of the currently reported red light‐activated reactions, alongside the discussion of the mechanisms underlying various catalytic systems. While the majority of reactions are facilitated through photocatalysis by using visible light, red light photocatalysis offers distinct advantages, which are attributable to the intrinsic properties of long‐wavelength light. The lower energy associated with red light reduces health risks and enhances tissue penetration depth, making red light photocatalysis a promising and powerful tool for applications in biochemical environments. Additionally, in longer‐wavelength regions, the activation of photocatalysts by low‐energy photons results in narrower redox windows. This phenomenon facilitates more precise control over chemical processes, allowing only specific reactions to occur under defined conditions.

However, red or NIR photocatalysis exhibits certain limitations. First, while the narrow redox windows associated with long‐wavelength light enhance the selectivity of reactions, they also present a challenge. Specifically, the range of reactions is constrained by these redox windows in the absence of additional metal catalysts or upconversion processes. Furthermore, the optimization strategies for photosensitizers, particularly for catalytic reactions in complex biological environments such as living cells or tumors, remain underdeveloped. Advancements in precise molecular modifications and structural optimizations, aimed at enhancing photocatalytic efficiency, solubility, and catalyst stability, are expected to improve the specificity of photocatalytic processes within these complex systems. The distinct reactions only facilitated by red light remain in the early stages of exploration. Moreover, asymmetric synthesis represents a primary goal in the field of chemistry, as stereoisomeric compounds with various enantiomers exhibit varying biological activities. Nevertheless, the development of asymmetric photocatalysis driven by red or NIR light irradiation remains limited. Various methodologies can be employed to overcome these constraints. One such approach involves the utilization of a dual red light‐photoredox/metal catalytic system featuring chiral ligands, which has the potential to yield asymmetric molecules. Moreover, the translation of red and NIR light‐mediated photocatalysis, such as PDT, into clinical applications continues to face challenges and limitations. Firstly, while red and NIR light exhibit superior tissue penetration compared to blue or UV light, their intensity diminishes as penetration depth increases due to scattering and absorption by endogenous chromophores, including hemoglobin, melanin, and lipids. Secondly, the stability of photocatalysts or photosensitizers is a critical factor, as they may undergo deactivation due to enzymatic degradation or pH fluctuations in the biological environment. Additionally, heterogeneous photocatalysts or photosensitizers, such as TiO₂ or COFs, are prone to aggregation within physiological systems, leading to reduced catalytic efficiency. At last, the potential toxicity of these materials poses a concern, as their prolonged retention in the body may induce inflammation or organ damage. Addressing these challenges is essential for the successful clinical implementation of red and NIR light‐mediated photocatalysis in therapeutic applications. This review presents an exposition on the rapid expansion of red or NIR photocatalysis. We believe that this review will assist researchers not only in the field of organic synthesis but also those in the disciplines of medicine and material science. Further advancements and broader applications of red or NIR light‐mediated photocatalysis are expected, thereby continually attracting much more attention in this field.

## Conflict of Interests

The authors declare no conflict of interest.

## Data Availability

The data that support the findings of this study are available from the corresponding author upon reasonable request.
